# Pain and pain tolerance in whiplash‐associated disorders: A population‐based study

**DOI:** 10.1002/ejp.819

**Published:** 2015-11-16

**Authors:** S.M. Myrtveit, J.C. Skogen, B. Sivertsen, Ó.A. Steingrímsdóttir, A. Stubhaug, C.S. Nielsen

**Affiliations:** ^1^ Department of Clinical ScienceUniversity of BergenNorway; ^2^ Division of Mental HealthNorwegian Institute of Public HealthBergenNorway; ^3^ Alcohol and Drug Research Western NorwayStavanger University HospitalStavangerNorway; ^4^ Uni HealthUni ResearchBergenNorway; ^5^ Department of PsychiatryHelse Fonna HFHaugesundNorway; ^6^ Division of EpidemiologyNorwegian Institute of Public HealthOsloNorway; ^7^ Department of Pain Management and ResearchOslo University Hospital and Faculty of MedicineUniversity of OsloNorway; ^8^ Division of Mental HealthNorwegian Institute of Public HealthOsloNorway

## Abstract

**Background:**

Pain is a cardinal symptom in individuals with whiplash‐associated disorders (WAD). We aimed to compare pain characteristics between individuals with WAD and individuals reporting chronic pain from other causes, and to determine whether potential differences were accounted for by experimental pain tolerance.

**Methods:**

Data from the 6th Tromsø Study (2007–2008, *n* = 12,981) were analysed. The number of painful locations was compared between individuals with WAD and individuals reporting chronic pain from other causes using negative binomial regression, pain frequency using multinomial logistic regression and pain intensity using multiple linear regression. Differences in experimental pain tolerance (cold pressor test) were tested using Cox regression; one model compared individuals with WAD to those with chronic pain from other causes, one compared the two groups with chronic pain to individuals without chronic pain. Subsequently, regression models investigating clinical pain characteristics were adjusted for pain tolerance.

**Results:**

Of individuals with WAD, 96% also reported other causes for pain. Individuals with WAD reported a higher number of painful locations [median (inter‐quartile range): 5 (3.5–7) vs. 3 (2–5), *p* < 0.001] and higher pain intensity (crude mean difference = 0.78, *p* < 0.001) than individuals with chronic pain from other causes. Pain tolerance did not differ between these two groups. Compared to individuals without chronic pain, individuals with WAD and individuals with chronic pain from other causes had reduced pain tolerance.

**Conclusions:**

Individuals with WAD report more additional causes of pain, more painful locations and higher pain intensity than individuals with chronic pain from other causes. The increased pain reporting was not accounted for by pain tolerance.

1

**What's already known about this topic?**

- After whiplash injuries some individuals develop substantial pain.

**What does this study add?**

- Individuals with whiplash‐associated disorders report a greater number of painful locations and higher pain intensity than individuals with chronic pain due to other conditions.
- Individuals with whiplash‐associated disorders almost always report additional causes for pain.
- The increased pain report in individuals with whiplash‐associated disorders compared to individuals with other pain cannot be accounted for by differences in experimental pain tolerance.

## 1 Introduction

The term whiplash‐associated disorders (WAD) refers to varying clinical manifestations reported after whiplash injuries (Spitzer, 1995). Some individuals develop chronic symptoms (Barnsley et al., 1994; Lovell and Galasko, 2002; Sterner and Gerdle, 2004; Rebbeck et al., 2006; Carroll et al., 2008; Matsumoto et al., 2010), and as expected, head and neck pain are among the symptoms most commonly reported in WAD (Gargan and Bannister, 1990; Squires et al., 1996; Berglund et al., 2001; Miettinen et al., 2002, 2004). However, pain in areas further from the neck, e.g. lower back, arms and legs is also reported (Berglund et al., 2001; Ferrari et al., 2005). This widespread pain profile resembles pain patterns seen in other chronic pain conditions, like fibromyalgia (Wenzel et al., 2009) and rheumatoid arthritis (Leavitt et al., 1986).

One explanation for the widespread pain in WAD could be sensitization of the somatosensory system (Sterling et al., 2011b; van Wilgen and Keizer, 2012). Central sensitization, defined as increased responsiveness of nociceptive neurons in the central nervous system, can result in widespread hyperalgesia which has been observed in a number of chronic pain conditions (Latremoliere and Woolf, 2009; Woolf, 2011). In small clinical studies, muscular hyperexitability, a larger area with referred pain (Johansen et al., 1999) and reduced pain thresholds (Curatolo et al., 2001; Scott et al., 2005) have been found in individuals with WAD. These findings are yet to be replicated in larger, population‐based studies. Increased knowledge of the pain characteristics in WAD, and the mechanisms behind it, can potentially enable better treatment for these patients.

The aim of the study was threefold: Firstly, we aimed to describe pain characteristics (number of painful locations, pain frequency and pain intensity) in individuals with WAD and in individuals with chronic pain from other causes in a large, population‐based sample. We also aimed to compare these pain characteristics, as well as total number of causes for pain reported, between individuals with WAD and individuals with chronic pain from other causes.

Secondly, we aimed to investigate pain tolerance in individuals with WAD and individuals with chronic pain from other causes – both compared to each other and compared to individuals with no chronic pain.

Thirdly, if any differences in pain characteristics were found between individuals with WAD and individuals with chronic pain from other causes, we aimed to determine whether these were related to experimental pain tolerance, a suggested indicator of central sensitization.

## 2 Materials and methods

### 2.1 Data

**Figure 1:**
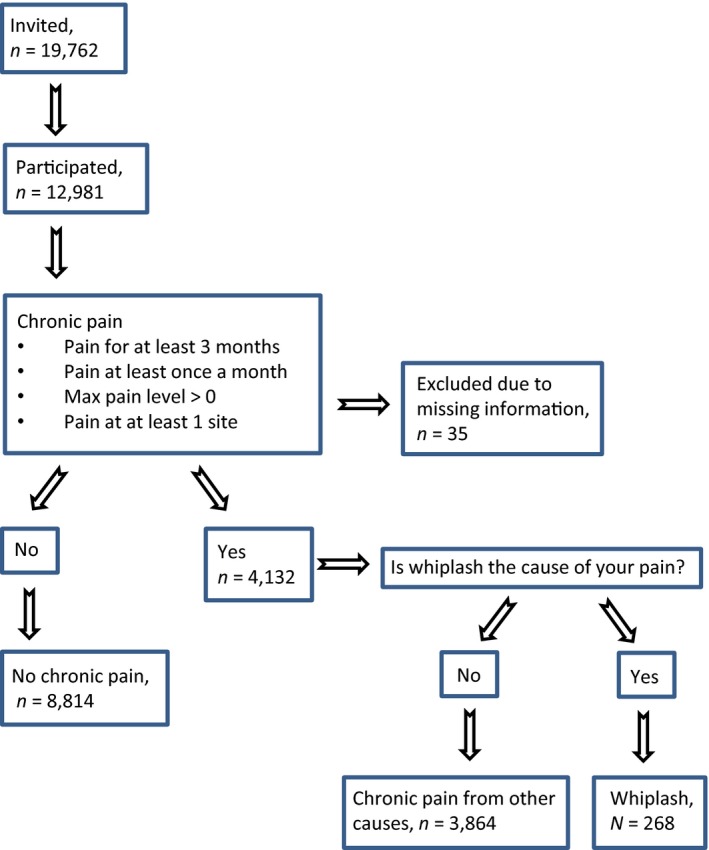
Grouping variable, *n* = 12,964, The 6th Tromsø Study.

Data from the sixth wave of the Tromsø Study (the 6th Tromsø Study) were analysed. In 2007–2008, a total of 12,981 individuals attended a cross‐sectional survey and medical examination in Tromsø municipality, northern Norway (see Fig. 1). All inhabitants from 40 to 42 years and from 60 to 87 years were invited to participate. In addition, 10–40% random samples of other age groups over 30 years of age were invited, adding up to a total of 19,762 invited individuals. The overall response rate was 66%. Detailed information on recruitment procedures, response rates and sample composition has been published previously (Jacobsen et al., 2012).

#### 2.1.1 Case definition – WAD and chronic pain from other causes

Participants were asked if they suffered from persistent pain that had lasted for 3 months or longer (screening question for chronic pain). They were then asked how long they had experienced this pain, how often they experienced it and what the maximum pain intensity was on a 0–10 numeric rating scale (NRS, with the anchors ‘No pain’ and ‘Worst pain imaginable’). Participants were also presented with a list of body regions and could indicate where they experienced pain; head/jaw, neck, back, shoulder, arm/elbow, hand, hip, thigh/knee/lower leg, ankle/foot, chest/breast, stomach, genitalia/reproductive organs, skin or other location.

Individuals were characterized as having chronic pain if they answered yes to the screening question of chronic pain, and/or reported: (1) pain for at least 3 months; (2) pain at least once a month; (3) maximum pain intensity above zero; and (4) at least one pain site (see Fig. 1).

Participants were also asked what they believed caused their pain. Fifteen non‐exclusive response options, including whiplash, were provided. Our sample was divided into three groups: individuals reporting whiplash as a cause of chronic pain, individuals reporting chronic pain from other causes and individuals reporting no chronic pain. Individuals reporting whiplash will in the following be referred to as individuals with WAD. Due to missing information, 35 participants could not be grouped with regard to chronic pain, and were excluded.

As participants could indicate more than one cause of pain, the WAD group includes both subjects reporting whiplash as the only cause of pain as well as subjects reporting whiplash and other causes. All individuals reporting whiplash as a cause of pain were included for the analyses, regardless of whether they also reported other causes.

#### 2.1.2 Number of painful locations, pain frequency and pain intensity

Participants were presented with a list of 15 locations (described above) and could indicate all painful areas. The number of locations reported as painful by each individual was captured in a count variable.

Participants indicated how often they experienced pain; ‘every day’, ‘once a week or more’, ‘once a month or more’ and ‘less than once a month’. Due to low *n* (*n* = 357 and *n* = 67 respectively), the last two categories were grouped together as ‘less than once a week’. This group was assigned the value 1, ‘once a week or more’ was assigned 2 and ‘every day’ was assigned 3.

Maximal pain intensity was also recorded (scale described above).

#### 2.1.3 Pain tolerance

Pain tolerance was tested with the cold pressor test (Chen et al., 1989), conducted using a 3 °C circulation water bath (Julabo PF40‐HE, JULABO Labortechnik GmbH, Germany) connected to a 13‐L external container with a flow of 22 L/min. Participants lowered their dominant hand and wrist into the bath for a maximum of 106 s. Endurance time (time participants managed to keep hand submerged in water) was recorded.

When data were collected, participants were asked to meet on a given day but were free to arrive at any time during that day. This led to peak hours which in combination with periods of sickness among the study staff made it impossible to examine all subjects. In such situations, the staff was told to prioritize individuals below 60 years of age due to the lower sampling rate in the younger age groups. In total, 2479 participants were not tested, and were excluded from analyses investigating pain tolerance. Among tested individuals, 2.0% reported WAD and 29.5% reported chronic pain from other causes, among those not tested 2.2% reported WAD and 31.3% reported chronic pain from other causes (*p* = 0.194).

#### 2.1.4 Background variables

Age and gender was obtained from the Norwegian Tax Administration. Subjects were grouped according to self‐reported marital status as single, married, widow/widower, separated/divorced or in a partnership. Based on self‐reported educational attainment, participants were grouped as having completed: (1) primary or secondary school only; (2) high school or vocational school; and (3) college or university.

Psychological distress was measured using the Hopkins Symptom Checklist, 10‐item version (SCL‐10). The SCL‐10 is a short version of the SCL‐25 and the SCL‐90 and has been found to correlate well with, and perform almost as good as, the longer lists (Rosen et al., 2000; Strand et al., 2003). Based on findings from previous studies (Stabell et al., 2013), a cut‐off of 1.85 for the mean SCL‐10 score (range 1–4, higher value representing more psychological distress) was used to distinguish those with high psychological distress from others.

### 2.2 Statistical analyses

The background variables age, gender, education and psychological distress were described using proportions (and chi‐square tests) and medians and inter‐quartile range (IQR, Mann–Whitney tests) as appropriate.

Number of painful locations, pain frequency, pain intensity as well as number of causes for pain reported were described in individuals with WAD and in individuals with chronic pain from other causes, and compared between these two groups. As these variables were not normally distributed, medians and IQR were reported for intensity and number of painful locations. For pain frequency, proportion reporting pain at each frequency was reported. Significance testing was performed using the Mann–Whitney test. Percentage within each group reporting pain in different locations was compared between individuals with WAD and individuals with chronic pain from other causes using chi‐square tests.

Pain tolerance was investigated using Cox proportional hazard models. Endurance time (time participants managed to keep hand submerged in water) was entered as survival time. If maximal time (106 s) was reached, data were defined as censored. If the participant aborted the test (withdrew hand) before maximal time, data were defined as failure. Results are reported as hazard ratios (HR) with 95% confidence intervals (CI). The HRs indicate the proportional hazard (for one group compared to another) at any time point during the test, to abort the pain stimulus.

Cox proportional hazard models comparing individuals with WAD to individuals reporting chronic pain from other causes were run. The analyses were adjusted for age, gender, education, marital status and psychological distress. The co‐variables were added to the model one at the time, apart from education and marital status that were added together.

Cox proportional hazard models comparing the two groups reporting chronic pain to those reporting no chronic pain were also conducted and adjusted as above. For both Cox regression models, the assumption of proportional hazards was investigated.

Associations between pain characteristics and the two chronic pain groups were assessed using negative binomial regression analyses (number of painful locations, over‐dispersed data), multiple linear regression models (pain intensity) and multinomial logistic regression (pain frequency, ‘less than once a week’ was set as reference category). In order to investigate whether differences in pain characteristics could be explained by differences in pain tolerance, analyses were adjusted for pain tolerance (cold pressor endurance time). Analyses were also adjusted for age, gender, education, marital status and psychological distress. The order the co‐variables were added to the model is described in Table 2. Assumptions regarding the linear regression model were assessed by means of regression diagnostics.

To account for missing data, 10 new data sets were created using multiple imputation of missing values. These data sets were analysed and results combined to produce point estimates and confidence intervals. As the findings did not differ substantially between the original and the imputed data sets, findings from the original set are presented.

Stata 12 was used for all analyses (StataCorp 2011).

## 3 Results

### 3.1 Description of groups and anthropometric, demographical and clinical characteristics

In our sample of 12,946 individuals, the median age was 59 years (IQR = 46–67) and 53.4% were women.

As detailed in Fig. 1, 4132 individuals fulfilled the criteria for chronic pain. Of these, 268 individuals reported whiplash as a cause of chronic pain (2.1% of total study sample). Chronic pain from causes other than whiplash was reported by 3864 individuals (29.9% of total study sample). A total of 8814 individuals reported no chronic pain (68.1% of total study sample). Anthropometric, demographical and clinical characteristics for each group are described in Table 1.

**Table 1:** Anthropometric, demographic and clinical characteristics in participants with WAD, chronic pain from other causes and no chronic pain, *n* = 12,946, The 6th Tromsø Study

	WAD, *n* = 268	Chronic pain from other causes, *n* = 3864	No chronic pain, *n* = 8814	*p*‐values^a^
Female	59.7%	62.0%	49.4%	0.447
Age at participation (median (IQR))	58.5 (46–64)	59 (47–66)	59 (46–67)	0.189
Education				0.131
Primary/secondary school	37.3%	32.8%	28.0%	
High school/vocational school	33.6%	34.9%	32.2%	
University/college	29.1%	32.3%	39.8%	
Psychological distress, HSCL‐10	27.4%	14.3%	4.9%	<0.001
Pain characteristics				
Number of pain locations (median (IQR))	5 (3.5–7)	3 (2–5)	N/A	<0.001
Pain intensity (median (IQR))	8 (7–9)	8 (6–8)	N/A	<0.001
Pain frequency				0.461
Every day	50.8%	55.1%	N/A	
Once a week or more	41.5%	34.1%	N/A	
Less than once a week	7.8%	10.8%	N/A	
Number of causes (median (IQR))	2 (1–3)	1 (1–2)	N/A	<0.001

HSCL‐10: Mean of ten item Hopkins Symptom Checklist (range 1–4), cut‐off for dichotomous variable; 1.85. Maximum pain intensity was measured on a NRS from 0 to 10, anchors ‘No pain’ and ‘Worst pain imaginable’. IQR, inter‐quartile range; WAD, whiplash‐associated disorders.

a *p*‐values for comparison between individuals with WAD and individuals with chronic pain from other causes derived using Mann–Whitney test for all comparisons apart from gender and psychological distress (chi‐square tests).

### 3.2 Painful locations

**Figure 2:**
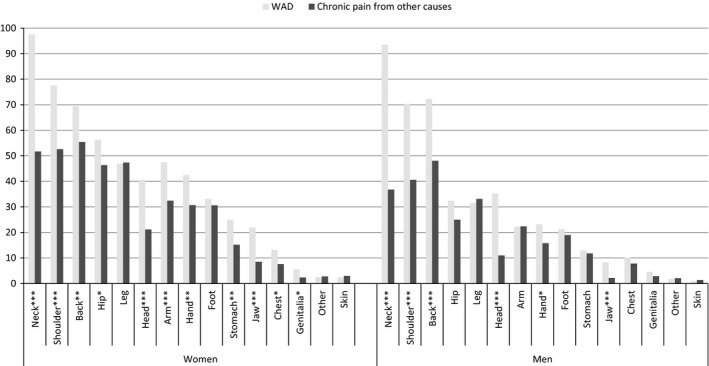
Percentage of participants within each group (WAD and chronic pain from other causes) reporting pain in each location. Analyses stratified by gender, *n* = 4132, The 6th Tromsø Study. WAD, whiplash‐associated disorders.

As shown in Fig. 2, both men and women with WAD were more likely to report pain in the neck, shoulder, back, head and jaw than individuals reporting chronic pain from other causes (all *p* < 0.01). Women with WAD also reported pain in the hip, arm, hand, stomach, chest and genitalia more often (all *p* < 0.05 compared to women with chronic pain from other causes).

### 3.3 Pain intensity and frequency

Reported pain intensity was higher among individuals with WAD than among individuals with chronic pain from other causes [median = 8, IQR: (7–9) vs. median = 8, IQR: (6–8), *p* < 0.001]. As detailed in Table 2, the crude mean difference in pain intensity between the two groups was 0.78 (95% CI: 0.49–1.08, *p* < 0.001), a small but statistically significant difference. Distribution of residuals was assessed by inspection of a series of scatter plots of residuals and each of the explanatory variables and of residuals and fitted values. Histograms and QQ plots were inspected to assess normality of the residuals. None of the plots gave rise to concern.

**Table 2:** Number of painful locations, pain intensity and pain frequency compared between individuals with WAD and individuals reporting chronic pain from other causes, crude and adjusted analyses, The 6th Tromsø Study

	WAD compared to chronic pain from other causes
Number of painful locations (Negative binomial regression)	
Incidence Rate Ratios (IRR) (95%CI)	
Model 1	1.45 (1.34–1.57)^a^
Model 2	1.48 (1.37–1.60)^a^
Model 3	1.47 (1.36–1.59)^a^
Model 4	1.46 (1.36–1.58)^a^
Model 5	1.39 (1.28–1.50)^a^
Pain intensity (Multiple linear regression)	
Mean difference (95%CI)	
Model 1	0.78 (0.49–1.08)^a^
Model 2	0.78 (0.49–1.07)^a^
Model 3	0.77 (0.48–1.06)^a^
Model 4	0.75 (0.46–1.03)^a^
Model 5	0.68 (0.38–0.98)^a^
Pain frequency (Multinomial logistic regression)	
Relative risk ratio (RRR) (95%CI)^b^	
Less than once a week	Ref.
Once a week or more	1.53 (0.90–2.58)
Every day	1.20 (0.72–2.01)

Mean difference = mean(whiplash)‐mean(other cause of chronic pain).

CI = confidence intervals.

HSCL‐10: Mean of the ten item Hopkins Symptom Check List (range 1–4).

Maximum pain intensity was measured on a NRS from 0 to 10, anchors “No pain” and “Worst pain imaginable”.

Pain frequency: coded: 1 = “less than once a week”, 2 = “once a week or more”, 3 = “every day”.

a *p *< 0.001.

b As no differences between the two groups were found for pain frequency, adjustments were not conducted.

Model 1: crude.

Model 2: adjusted for age and gender.

Model 3: adjusted for age, gender and cold pressor endurance time.

Model 4: adjusted for age, gender, cold pressor endurance time, education and marital status.

Model 5: adjusted for age, gender, cold pressor endurance time, education, marital status and psychological distress (HSCL‐10).

Reported pain frequency did not differ between the two groups (Table 2).

### 3.4 Number of painful locations

As detailed in Table 1, the median number of painful locations reported was higher among individuals with WAD than among individuals with chronic pain from other causes [median = 5, IQR: (3.5–7) vs. median = 3, IQR: (2–5), *p* < 0.001]. Individuals with WAD had a higher risk of reporting more painful locations than individuals with chronic pain from other causes (crude; incidence rate ratios (IRR) = 1.45, 95% CI: 1.34–1.57, *p* < 0.001) (see Table 2 for adjusted estimates).

### 3.5 Number of causes for pain

Of the 268 individuals with WAD, only 10 individuals reported whiplash exclusively. Thus, 96.3% of individuals with WAD reported additional causes for pain. Individuals with WAD also reported causes like long‐term strain (47.4%), herniated disc (25.4%), headache/migraine (23.9%) and fibromyalgia (16.0%). As detailed in Table 1, the median number of other causes of chronic pain for individuals with WAD was 2 [IQR: (1–3)], while the median number of causes indicated by individuals with chronic pain from other causes was 1 [IQR: (1–2)], *p* < 0.001.

### 3.6 Experimental pain tolerance

The cold pressor test was aborted before the maximum time of 106 s by 29.7% of individuals reporting no chronic pain, by 39.3% of individuals with WAD and by 35.8% of those reporting chronic pain from other causes.

The experimental pain tolerance (cold pressor endurance time) did not differ between individuals with WAD and individuals with chronic pain from other causes, as presented in Table 3: The HR for hand withdrawal before maximum immersion time among individuals with WAD compared to individuals with chronic pain from other cause was 1.13 (95% CI 0.91–1.41, *p* = 0.279) in the crude model, and 1.09 (95% CI: 0.85–1.39, *p* = 0.492) in the fully adjusted model. The test of proportional hazards gave no reason to reject the hypothesis that the hazards are proportional (crude model: ρ: −0.010, χ^2^: 0.13, *p*‐value: 0.720).

**Table 3:** Hazard ratio (HR) for hand withdrawal during the cold pressor test in participants reporting WAD compared to those with chronic pain from other causes, crude and adjusted analyses shown, The 6th Tromsø Study

	Chronic pain from other causes	WAD HR (95% CI)
Crude estimates	Ref.	1.13 (0.91–1.41)
Adjusted for age	Ref.	1.13 (0.90–1.41)
Adjusted for age, gender	Ref.	1.16 (0.93–1.45)
Adjusted for age, gender, education and marital status	Ref.	1.14 (0.91–1.42)
Adjusted for age, gender, education, marital status and HSCL	Ref.	1.09 (0.85–1.39)

HSCL = Mean of the ten item Hopkins Symptom Check List (Psychological distress), range 1–4. CI: confidence intervals.

Compared to individuals with no chronic pain, pain tolerance was lower in both individuals with WAD (crude HR = 1.44, 95% CI: 1.16–1.79, *p* = 0.001) and individuals reporting chronic pain from other causes (crude HR = 1.27, 95% CI: 1.18–1.37, *p* < 0.001). These differences were still significant after adjusting for age, gender and education, but became non‐significant when also adjusting for psychological distress (WAD: HR = 1.19, 95% CI: 0.94–1.51, *p* = 0.149, chronic pain from other causes: HR = 1.08, 95% CI: 0.99–1.17, *p* = 0.072). Test of proportional hazards showed violation of the proportional hazards assumption for the overall crude model, *p* = 0.034. Specifically, the proportional hazards assumption held for WAD compared to no chronic pain (ρ: −0.02 *p* = 0.239), but not for chronic pain from other causes compared to no chronic pain (ρ: −0.04, *p* = 0.015).

### 3.7 Pain tolerance and differences in pain characteristics

As detailed in Table 2, adjusting for pain tolerance (cold pressor endurance time) did not substantially change the estimated association for neither number of painful location nor pain intensity. As no significant differences in pain frequency were found, these analyses were not adjusted for cold pressor endurance time.

## 4 Discussion

### 4.1 Summary of findings

In this population‐based study, 96% of individuals reporting whiplash as a cause of pain also report other causes of chronic pain. Individuals with WAD were more likely to report pain in both proximal and distal body parts than individuals with chronic pain from other causes. They also report a higher total number of painful locations and slightly higher pain intensity. These differences were not accounted for by differences in pain tolerance. Pain tolerance did not differ between individuals with WAD and individuals with chronic pain from other causes. However, compared to individuals reporting no chronic pain both groups with chronic pain had reduced pain tolerance.

### 4.2 Interpretation of findings

In our study, only 10 of the individuals reporting whiplash as a cause of chronic pain, reported whiplash as their *only* cause. We are thus not investigating isolated whiplash‐related pain, rather whiplash pain in comorbidity with pain from other causes. Theoretically it would be interesting to investigate pain and pain tolerance in individuals with WAD only. However, individuals with WAD are known to suffer from a wide range of symptoms from different body regions (Myrtveit et al., 2012), and individuals reporting somatic diagnoses like diabetes, osteoporosis or fibromyalgia are at increased risk of both developing chronic whiplash (Myrtveit et al., 2013) and of not recovering from it (Myrtveit et al., 2014). Together, this previous research and our findings suggest that WAD almost always co‐occurs with other pain and non‐pain conditions. This comorbidity is in itself interesting, and one might discuss how common suffering from chronic pain from only whiplash really is. If individuals with WAD almost always suffer from other pain conditions as well, the clinical value of studying whiplash‐related pain in isolation would be limited.

Individuals with WAD reported a higher number of pain sites and slightly higher pain intensity than individuals with chronic pain from other causes. We investigated whether this could be explained by differences in pain tolerance. Reduced pain tolerance might be an expression of central sensitization. The central nervous system is plastic and repeated stimulation may lead to habituation (decreased response) or sensitization (increased response) (Eriksen and Ursin, 2004). Central sensitization, with attenuated anti‐nociceptive mechanisms (Meeus et al., 2008) and/or overactive pain facilitating pathways (Latremoliere and Woolf, 2009), is thought to be important in many chronic (pain) conditions (Woolf, 2011), including fibromyalgia and chronic fatigue syndrome (Meeus and Nijs, 2007). Central sensitization has also been suggested to play a role in the development and maintenance of chronic pain after whiplash injuries (Sterling et al., 2011b).

Compared to individuals reporting no chronic pain, reduced pain tolerance was found both in individuals with WAD and in individuals reporting chronic pain from other causes. Previous studies have found increased pain sensitivity in chronic pain conditions like irritable bowel syndrome (Stabell et al., 2013), fibromyalgia (Petzke et al., 2003) and osteoarthritis (Bajaj, Bajaj et al., 2001). Also, individuals with WAD have been shown to have reduced pain threshold (Curatolo et al., 2001; Scott et al., 2005), muscular hyperexitability and larger areas with referred pain (Johansen et al., 1999). The importance of altered pain processing is further underlined by prospective studies showing that reduced pain tolerance after whiplash injuries is associated with non‐recovery and disability (Kasch et al., 2005; Sterling et al., 2011a). The decreased pain tolerance found in individuals with WAD compared with individuals reporting no chronic pain might therefore be part of the reason individuals with WAD report more symptoms than the general population. However, differences in pain tolerance were no longer significant after controlling for psychological distress.

As no significant difference in pain tolerance was found between individuals with WAD and individuals reporting chronic pain from other causes, it might seem that the reason for increased pain reporting in WAD relative to other pain conditions lies elsewhere. In our study, psychological distress was the only covariate somewhat changing the associations with pain characteristics between individuals with WAD and individuals with chronic pain from other causes (Table 2). There is reason to believe that pain and mental health is tightly interwoven (Korff and Simon, 1996). As such, the impact of including psychological distress in our models could be related to pain resulting in higher levels of depression (Fishbain et al., 1997). It should, however, also be noted that psychosocial factors might affect pain and outcome after whiplash injuries (Sterling et al., 2011b) – as in other pain conditions (Pincus et al., 2002). Previous research has found increased risk of developing WAD among individuals reporting symptoms of anxiety (Myrtveit et al., 2013), anxiety and depression (Mykletun et al., 2011) and mental impairment (Wenzel et al., 2012) before the injury. Symptoms of anxiety is also associated with non‐recovery from WAD (Myrtveit et al., 2014).

### 4.3 Strengths and limitations

This study is cross‐sectional and no conclusions on causality can be drawn; WAD and other types of chronic pain could have affected participants’ pain tolerance – and pain tolerance could have affected the risk of chronic pain.

The overall response rate in this study was 66% (Jacobsen et al., 2012). Over the last decades, participation in population‐based studies has been declining (Krokstad et al., 2013). In general, individuals who participate in studies are healthier (Knudsen et al., 2010) and we might expect that individuals with very severe WAD, of for instance WAD grade 4, would be less likely to participate. However, as these cases might be qualitatively different from lower WAD grades, they are often excluded from research (Spitzer, 1995; Kongsted et al., 2007). It has further been argued that the risk of biased results is larger for prevalence estimates of exposures and outcomes than for exposure‐outcome associations (Nilsen et al., 2009) and that the generalizability of associations often is sufficient even when distribution of measurements in the study population is different from the general population (Manolio and Collins, 2010). Still, selection bias might indeed have affected our results and care should be taken when generalizing the results to other populations.

The grouping variable and most co‐variables are based on self‐reported information with no medical information. A total of 35 participants did not provide enough information to be grouped with regards to pain, and were excluded from our analyses. Age, gender and psychological distress did not differ between these individuals and those that could be grouped with regard to pain (data not shown).

Experimental pain tolerance was tested using the cold pressor test. Multiple modalities of pain can be tested when investigating human pain, and different methods and modalities correlate poorly (Neziri et al., 2011). Hence, a different method could have given different results.

The results regarding number of pain locations should be interpreted with caution. The number of pain sites reported might vary with locations asked about; a list with relatively more locations from one body region might lead to increase of total number of locations reported in patients with pain in that specific region. Based on visual inspection of Fig. 2, the differences in pain reporting seem largest for areas close to the neck. More listed locations from lower extremities might have given smaller differences in number of pain sites.

As mentioned in the results section, in the Cox model comparing individuals with WAD and chronic pain from other causes to individuals with no chronic pain, the overall assumption of proportional hazards did not hold for chronic pain from other causes compared to no chronic pain (ρ: −0.04, *p* = 0.015). However, we investigated a large sample (*n* = 3089 individuals with chronic pain, *n* = 7164 with no chronic pain), and the value for ρ is small. *P*‐values are in part a function of sample size, and their usefulness declines as sample sizes grow very large – as the null hypothesis will almost never be exactly true. When including an interaction term between group and time in our model, the interaction term had a statistically significant but small HR (HR = 0.96, 95% CI: 0.96–0.96). The scaled Schonefeld residual plot showed a slope not far from flat with regards to time (plot not shown). There is thus reason to believe that though the proportional hazard assumption does not hold, the hazards do not deviate so much from being proportional that inference is impaired.

As most previous studies investigating pain sensitivity in individuals who have experienced whiplash injuries have been small clinical or experimental studies, a main strength of this study is the population‐based design and the large size, increasing generalizability and allowing comparison between groups and adjustment for co‐variables. However, the WAD group is much smaller (*n* = 268) compared to the other groups (chronic pain from other causes = 3864 and no chronic pain = 8814) possibly giving rise to issues related to the precision of the estimates.

## 5 Conclusion

In this population‐based study, 96% of individuals with WAD reported additional causes for pain. Individuals with WAD also reported pain in a wide range of bodily locations, a higher number of painful locations and higher pain intensity than individuals with chronic pain from other causes. These differences were not accounted for by differences in pain tolerance.

## Author contributions

C.S.N., B.S., J.C.S. and S.M.M. designed the study. S.M.M. and J.C.S. conducted the analyses under supervision of Ó.A.S., C.S.N. and B.S. After discussion with all authors, S.M.M. wrote the first draft of the manuscript. All authors contributed to the final write‐up of the manuscript.
